# Framework for exploring the sensory repertoire of the human gut microbiota

**DOI:** 10.1128/mbio.01039-24

**Published:** 2024-05-17

**Authors:** Patricia A. Ross, Wenhao Xu, Ekaterina Jalomo-Khayrova, Gert Bange, Vadim M. Gumerov, Patrick H. Bradley, Victor Sourjik, Igor B. Zhulin

**Affiliations:** 1 Department of Microbiology, The Ohio State University, Columbus, Ohio, USA; 2 Translational Data Analytics Institute, The Ohio State University, Columbus, Ohio, USA; 3 Max Planck Institute for Terrestrial Microbiology, Marburg, Germany; 4 Center for Synthetic Microbiology (SYNMIKRO), Marburg, Germany; 5 Department of Chemistry, Philipps-University Marburg, Marburg, Germany; 6 Infectious Diseases Institute, The Ohio State University, Columbus, Ohio, USA; Massachusetts Institute of Technology, Cambridge, Massachusetts, USA

**Keywords:** signal transduction, gut microbiome, receptors, amino acids

## Abstract

Bacteria sense changes in their environment and transduce signals to adjust their cellular functions accordingly. For this purpose, bacteria employ various sensors feeding into multiple signal transduction pathways. Signal recognition by bacterial sensors is studied mainly in a few model organisms, but advances in genome sequencing and analysis offer new ways of exploring the sensory repertoire of many understudied organisms. The human gut is a natural target of this line of study: it is a nutrient-rich and dynamic environment and is home to thousands of bacterial species whose activities impact human health. Many gut commensals are also poorly studied compared to model organisms and are mainly known through their genome sequences. To begin exploring the signals human gut commensals sense and respond to, we have designed a framework that enables the identification of sensory domains, prediction of signals that they recognize, and experimental verification of these predictions. We validate this framework’s functionality by systematically identifying amino acid sensors in selected bacterial genomes and metagenomes, characterizing their amino acid binding properties, and demonstrating their signal transduction potential.

**IMPORTANCE:**

Signal transduction is a central process governing how bacteria sense and respond to their environment. The human gut is a complex environment with many living organisms and fluctuating streams of nutrients. One gut inhabitant, *Escherichia coli*, is a model organism for studying signal transduction. However, *E. coli* is not representative of most gut microbes, and signaling pathways in the thousands of other organisms comprising the human gut microbiota remain poorly understood. This work provides a foundation for how to explore signals recognized by these organisms.

## INTRODUCTION

Bacteria constantly monitor changes in their environment to ensure survival. Using various receptors, bacteria can detect changes in temperature, pH, osmolarity, concentration of oxygen, and numerous small molecules acting as environmental cues. Signal transduction pathways in bacteria control metabolism, differentiation, quorum sensing and biofilm formation, motility, virulence, and other processes (1, 2). Signal recognition at the sensor domain of receptors initiates signal transduction processes, leading to changes in gene expression, second messenger turnover, or protein-protein interactions. Hundreds of different sensor domains have been identified, but only a few of them are abundant (3). Signals that activate most of the bacterial signal transduction pathways remain unknown, which is considered as a major bottleneck in signal transduction research (4). Identifying signals for thousands of unstudied receptors by inferring from a handful of well-characterized homologs is difficult because sensory domains show large sequence divergence due to their rapid evolution (5, 6). Iterative computational and experimental approaches combined with the wealth of available genomic data offer new ways of exploring the sensory repertoire of many understudied organisms (7–9). The human gut, in particular, is a natural target of this line of research.

The gut is a complex, dynamic environment that supports the growth of thousands of microbial species. These microbes need to detect which host- and diet-derived nutrients are most abundant (10, 11) and how the availability of those nutrients varies over time (12, 13), as well as detect small molecules from other members of the community (14). Gut microbial activity has wide-ranging impacts on host health, affecting immunity (15, 16), insulin signaling (17–19), and drug metabolism (20–23), and has been associated with conditions as diverse as inflammatory bowel disease (24), heart disease (25–27), and liver cirrhosis (28, 29). This makes it particularly important to understand not only which microbes are present but also how they sense and integrate signals from their environment to produce changes in behavior. Currently, however, our ability to discover specific gut microbial signaling pathways is limited: while abundant gut microbes are phylogenetically distant from model organisms, much of what we know about their signaling pathways has been transferred from these model organisms based on coarse-grained sequence similarity.

To begin exploring the signals human gut commensals sense and respond to, we have designed a framework that enables the identification of sensory domains, prediction of signals that they recognize, and experimental verification of these predictions.

## RESULTS

**Fig 1:**
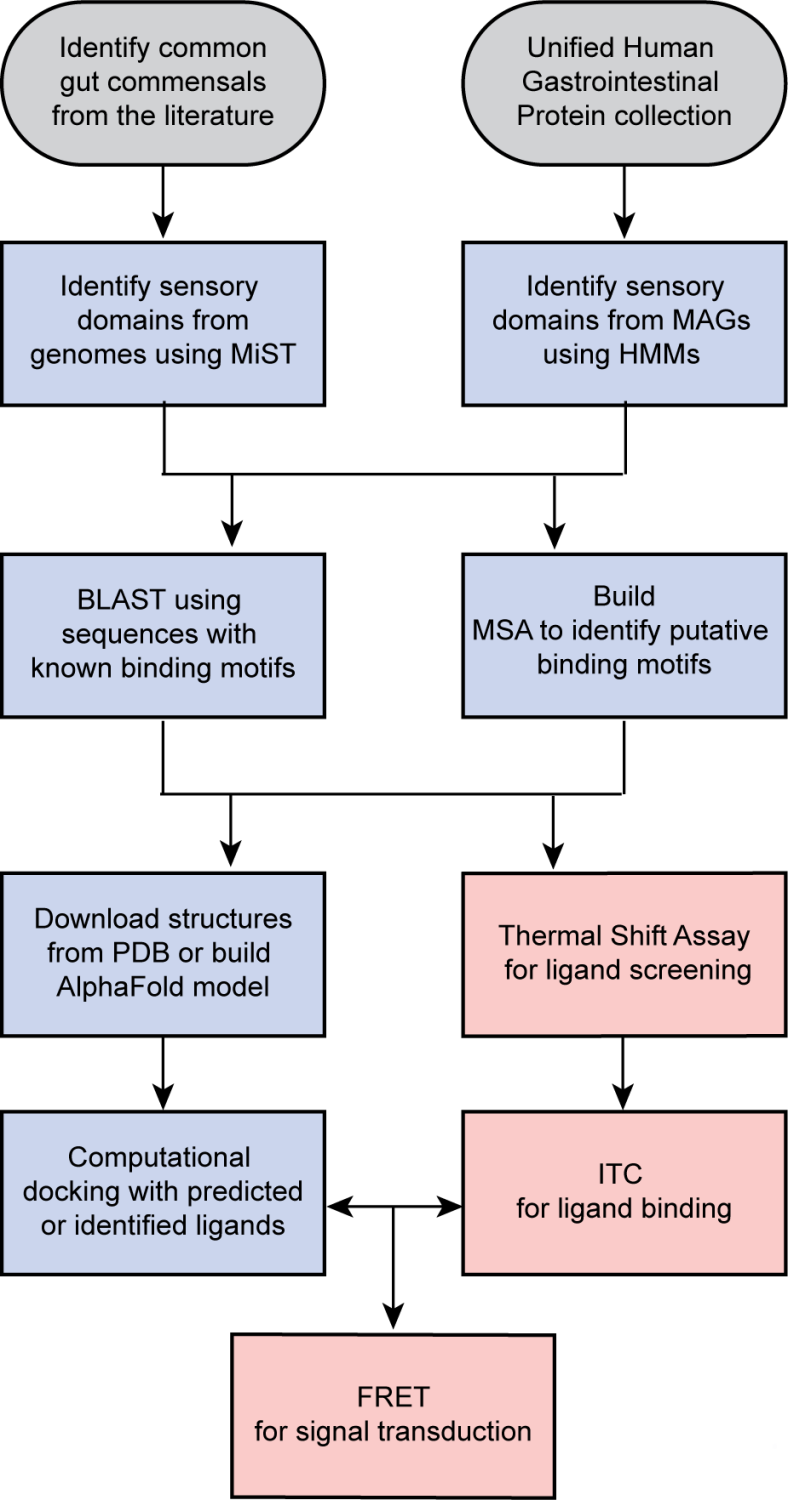
Workflow for identifying sensory domains in the human gut microbiome. Sources of information are shown in gray. Computational steps are shown in blue and experimental steps are shown in pink. FRET, Förster resonance energy transfer; HMM, hidden Markov model; ITC, isothermal titration calorimetry; MAG, metagenome assembled genome; MiST, Microbial Signal Transduction Database. Protein Data Bank (PDB).

Our first goal was to design an approach for identification of sensory domains in human gut microbiota. To achieve this goal, we needed access to genomes of gut commensals that primarily come from two sources: cultivated bacteria and metagenome data sets. To obtain genomes of cultivated gut commensals, we generated a list of bacteria that are commonly found in the healthy human gut microbiome and whose genomes were available for analysis. For mining metagenomic samples, we used the Unified Human Gastrointestinal Protein (UHGP) catalog, which has over 170 million protein sequences (30). These two sources of information required two different pipelines for genome mining, but once sensory domains are identified, both pipelines share downstream computational and experimental steps (Fig. 1).

### Curated list of bacterial commensals reported in the healthy human gut microbiome

Sixty species representing eight bacterial phyla were chosen based on extensive literature review (including published data sets) and reported relative abundance (Table 1; Data Set S1). Their genomes were accessed in the Microbial Signal Transduction Database (MiST) (31), which uses the National Center for Biotechnology Information (NCBI) RefSeq database (32) as a source of genomes. The NCBI BioSample database was used to confirm that the organism was isolated from the healthy human gut or related location (e.g., feces/stool). After the isolation source was confirmed, genomes were chosen using the most complete assembly level. Taxonomy information for each organism was taken from the Genome Taxonomy Database (33). The Unified Human Gastrointestinal Genome collection (UHGG) (30) was used to confirm common commensals identified during literature review. UHGG comprises a large set of metagenome-assembled genomes and isolated genomes from around the world and from various databases compiled in one place. The authors reported a geographical diversity score for each species by calculating the Shannon entropy of the proportion of samples containing that species on each continent. This score ranges from 0 (only observed on a single continent) to a maximum of ln(6) = 1.71 (equally observed across all six continents). Satisfactorily, most of the species that we identified in the literature review ranged between 1.0 and 1.5, indicating that they were found in a variety of geographical locations. By no means should our list be considered as the gold standard for typical human gut commensal bacteria. However, it provided us with a solid foundation for the first necessary step in our pipeline: identifying some of the common gut commensals to mine their genomes for sensory domains and signals that they recognize. In addition, our list can be used by others for other types of research that require initial genome mining of human gut commensals.

**TABLE 1:** Representative common human gut commensal bacteria

Organism^*a*^	Phylum	Genome accession no.	Biosample	References	UHGP quantity^*b*^
*Bifidobacterium adolescentis*	Actinomycetota	GCF_000817995.1	SAMN03273368	(30, 34–43)	2,454
*Roseburia faecis* (*Agathobacter faecis*)	Bacillota_A	GCF_001405615.1	SAMEA3545271	(30, 37, 39, 44)	2,855
(*Eubacterium*) *rectale* (*Agathobacter rectalis*)	Bacillota_A	GCF_001404855.1	SAMEA3545268	(30, 34, 36, 38, 39, 41–46)	7,406
*Blautia obeum*	Bacillota_A	GCF_000153905.1	SAMN00627103	(34–39, 43)	204
*Coprococcus eutactus*	Bacillota_A	GCF_000154425.1	SAMN00627069	(34, 39)	1,946
*Dorea longicatena*	Bacillota_A	GCF_000154065.1	SAMN00627086	(34, 35, 37, 39, 43)	800
*Faecalibacterium prausnitzii*	Bacillota_A	GCF_002586945.1	SAMN07764380	(30, 34–39, 41, 43, 46, 47)	7,687
*Fusicatenibacter saccharivorans*	Bacillota_A	GCF_001406335.1	SAMEA3545349	(30, 37, 39, 43)	3,227
*Eubacterium eligens* (*Lachnospira eligens*)	Bacillota_A	GCF_001405395.1	SAMEA3545343	(36, 37, 39, 42, 44)	3,111
(*Ruminococcus*) *torques (Mediterraneibacter torques*)	Bacillota_A	GCF_001405235.1	SAMEA3545275	(34, 35, 38, 39, 42)	1,614
*Roseburia intestinalis*	Bacillota_A	GCF_000156535.1	SAMN00008856	(34, 38, 39, 41, 42, 47)	1,041
*Ruminococcus bromii*	Bacillota_A	GCF_900101355.1	SAMN02910441	(30, 34–36, 38, 39, 41, 42, 44)	4,049
*Dialister invisus*	Bacillota_C	GCF_000433275.1	SAMEA3138551	(39, 42)	1,452
*Bacteroides dorei* (*Phocaeicola dorei*)	Bacteroidota	GCF_001640865.1	SAMN03737480	(30, 34, 36–39, 42, 43)	5,669
*Bacteroides fragilis*	Bacteroidota	GCF_000157015.1	SAMN02463689	(34, 36, 38, 39, 42, 43, 48–51)	1,246
*Bacteroides thetaiotaomicron*	Bacteroidota	GCF_001314975.1	SAMN03852684	(34, 36–39, 41–43, 50–54)	565
*Alistipes putredinis*	Bacteroidota	GCF_000154465.1	SAMN00000002	(30, 34, 39, 42, 44, 45)	4,979
*Parabacteroides distasonis*	Bacteroidota	GCF_013267615.1	SAMN11056474	(30, 34, 36–39, 41–43, 51)	4,638
*Sutterella wadsworthensis*	Pseudomonadota	GCF_000186505.1	SAMN02463869	(39, 42)	2,095
*Escherichia coli*	Pseudomonadota	GCF_000023365.1	SAMN02598470	(30, 34, 36–39, 41–44, 47, 48, 50, 55–60)	8,185
*Akkermansia muciniphila*	Verrucomicrobiota	GCF_009731575.1	SAMD00192834	(30, 34, 38, 39, 44, 48, 50, 55, 61, 62)	3,041

^*a*^ Species names commonly found in literature are shown, and current taxonomic assignments by Genome Taxonomy Database (when different from common names) are shown in parenthesis.

^*b*^ UHGP quantity is the number of genomes associated with that organism taken from the Genomes-all_metadata.tsv (link in Data Set S1).

### Identification of extracytoplasmic sensory domains in genomes of human gut commensals

In MiST, signal transduction proteins are identified via a built-in pipeline, which groups them into relevant functional categories (31). For the purpose of this study, we focused on extracytoplasmic sensory domains because they detect most of environmental signals. To identify these domains in cultivated human gut commensals listed in Data Set S1, we mined their genomes to collect protein sequences of chemoreceptors and sensor histidine kinases of the two-component regulatory systems as well as nucleotide cyclases, cyclic nucleotide phosphodiesterases, transcriptional regulators, and serine/threonine kinases and phosphatases of one-component systems (see Materials and Methods for details). The MiST pipeline enables identification of transmembrane helices and the overall membrane topology, which we used to collect all domains from these receptor proteins that were predicted to be extracytoplasmic. In ambiguous cases, the transmembrane topology predictor Phobius (63) was used to verify results obtained from MiST.

**Fig 2:**
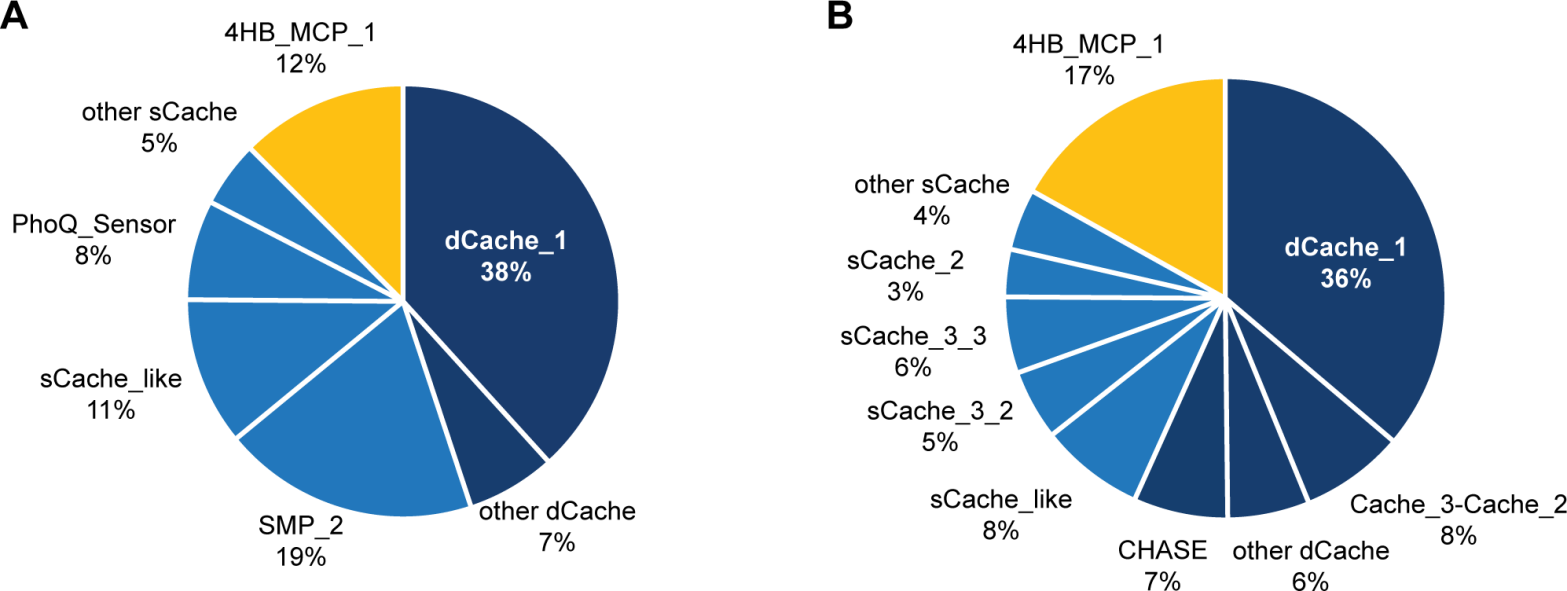
Extracytoplasmic sensory domains identified in the human gut microbiome. (**A**) Distribution of domains identified in genomes of cultivated human gut commensal bacteria. The underlying data are available as Data Set S2. (**B**) Distribution of domains identified in the UHGP catalog. The underlying data are available as Data Set S3. Families of the Cache superfamily (clan CL0165) are shown in blue: double-module Cache domain families in dark blue and single-module Cache domain families in light blue. A family of the 4HB_MCP superfamily (clan CL0457) is shown in yellow. Domain nomenclature and accession numbers are from the Pfam database.

When no domain was predicted by MiST in a region of >100 amino acid residues flanked by two transmembrane helices, we used a more sensitive profile-profile similarity search tool, HHpred (64) (with a probability threshold of >90%), to identify potential sensory domains. As a result, 741 sensory domains that belong to 15 families were identified in 60 genomes of cultivated human gut commensals (Data Set S2). The majority of identified sensory domains belong to the eukaryotic Ca^2+^ channels and bacterial chemoreceptors (Cache) domain superfamily (Fig. 2A), which was previously reported as the largest extracellular sensory domain superfamily in prokaryotes (65). Other identified domains belong to the four-helix bundle in methyl-accepting chemotaxis protein (4HB_MCP) superfamily, which was also previously identified as a ubiquitous sensory module in bacteria and archaea (66).

### Identification of extracytoplasmic sensory domains in human gut metagenomic data sets

Metagenomics has become the major approach to study microbiota (34, 35, 44, 45), and the metagenomics-driven UHGG database is one of the largest collections of assembled genomes from the human gut, including uncultivated human gut commensals. We used the UHGP catalog, which collates approximately 170 million protein sequences from more than 200,000 non-redundant UHGG genomes that represent nearly 5,000 prokaryotic species (30). Because these metagenomically assembled genomes are not available in MiST and identifying all signal transduction proteins in such a vast sequence space requires substantial computing and computational efforts, we chose a simplified strategy as our initial approach to mining the UHGP catalog. We used profile hidden Markov models (HMMs) from Pfam (which is now a part of the InterPro resource [67]) that correspond to all extracellular sensory domain families that we have identified in common cultivated human gut commensals (Data Set S2). Using these HMMs in HMMER searches, we scanned the entire UHGP catalog and identified >16,000 domains that belong to Cache and 4HB_MCP superfamilies (Fig. 2B; Data Set S3). Despite a substantial difference in size, the relative proportion of Cache and 4HB_MCP domains in two data sets (genomes of cultivated species vs metagenomically assembled genomes) was quite similar (Fig. 2).

### Identification and initial characterization of amino acid sensors from UHGP

**Fig 3:**
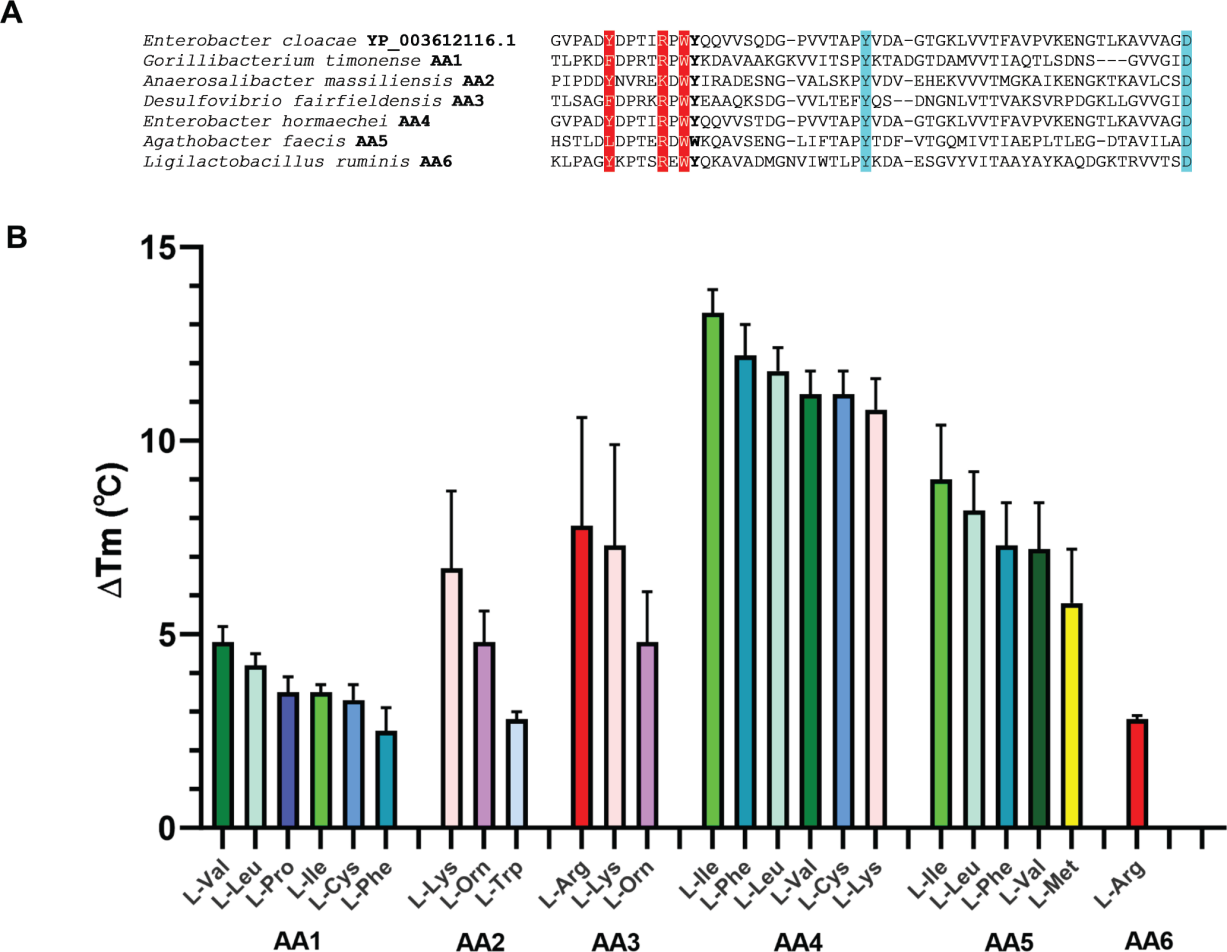
Representative predicted dCache_1AA domains and their putative ligands. (**A**) Multiple sequence alignment of the known dCache_1AA domain from *Enterobacter cloacae* chemoreceptor with six putative dCache_1AA domains from human gut commensals. (**B**) The putative ligands for six dCache_1AA domains. Thermal shift assays for six purified dCache_1AA domains (AA1–AA6) were performed in the presence of bacterial nitrogen sources from the Biolog screen plate PM3B. Except for the dCache_1AA domain AA4, all compounds that induced a shift in the midpoint of protein unfolding transition (Tm) of at least 2°C are presented. The complete list of positive ligands for the dCache_1AA domain AA4 is shown in Fig. S1. The data shown are the means and standard errors from three biological replicates conducted in triplicate.

To begin systematic exploration of signals recognized by thousands of sensory domains identified in the UHGP catalog, we focused on the identification of amino acid sensors. This was due to the reasons that (i) we have previously identified a universal amino acid binding motif in dCache_1 domains (7) and (ii) in our data sets, dCache_1 domains comprised the largest family group (Fig. 2). We used the dCache_1 domain sequence with flanking transmembrane helices (residues 9–294) from an *Enterobacter cloacae* chemoreceptor (NCBI accession number YP_003612116), which was previously identified as an amino acid sensor (7), as a query in a BLAST search against all dCache_1 domains identified from UHGP. Similar sequences from Data Set S2 and Data Set S3 and were then aligned (multiple sequence alignment is available at https://zenodo.org/records/10806258), and the amino acid binding motif, [YFWL]xxx[RK]xW[YFW][x ~ 13–17][YFW][x ~ 27–34]D, was identified in 539 of them (Data Set S4). We then selected six putative amino acid binding dCache_1 domains (termed AA1–AA6) from chemoreceptors representing four different bacterial phyla for experimental validation (Fig. 3A; Data Set S5). We synthesized and purified these domains *in vitro* and performed high-throughput thermal shift assay (68) using the commercially available compound array Biolog PM3B (see Materials and Methods), which contains all 20 common amino acids and 75 other compounds that were considered as nitrogen sources. Consistent with our prediction, all ligands that elicited significant changes in the melting temperature upon binding to these selected sensory domains were L-amino acids (Fig. 3B), although their exact specificity differed between domains. The AA4 domain showed the broadest amino acid specificity, binding not only the majority of proteinogenic L-amino acids but also a number of amino acid derivatives (Fig. 3B; Fig. S1A and B). The ligand spectrum for other five domains was limited to one or a few L-amino acids (Fig. 3B), and no other tested compounds from the PM3B set produced significant thermal shift. Notably, none of the tested domains, including AA4, showed binding to D-amino acids that were among the tested compounds. We thus confirmed that all these domains containing the dCache_1AA motif are indeed amino acid sensors, as suggested previously (7).

### Verification of amino acid binding by selected dCache_1AA domains

**Fig 4:**
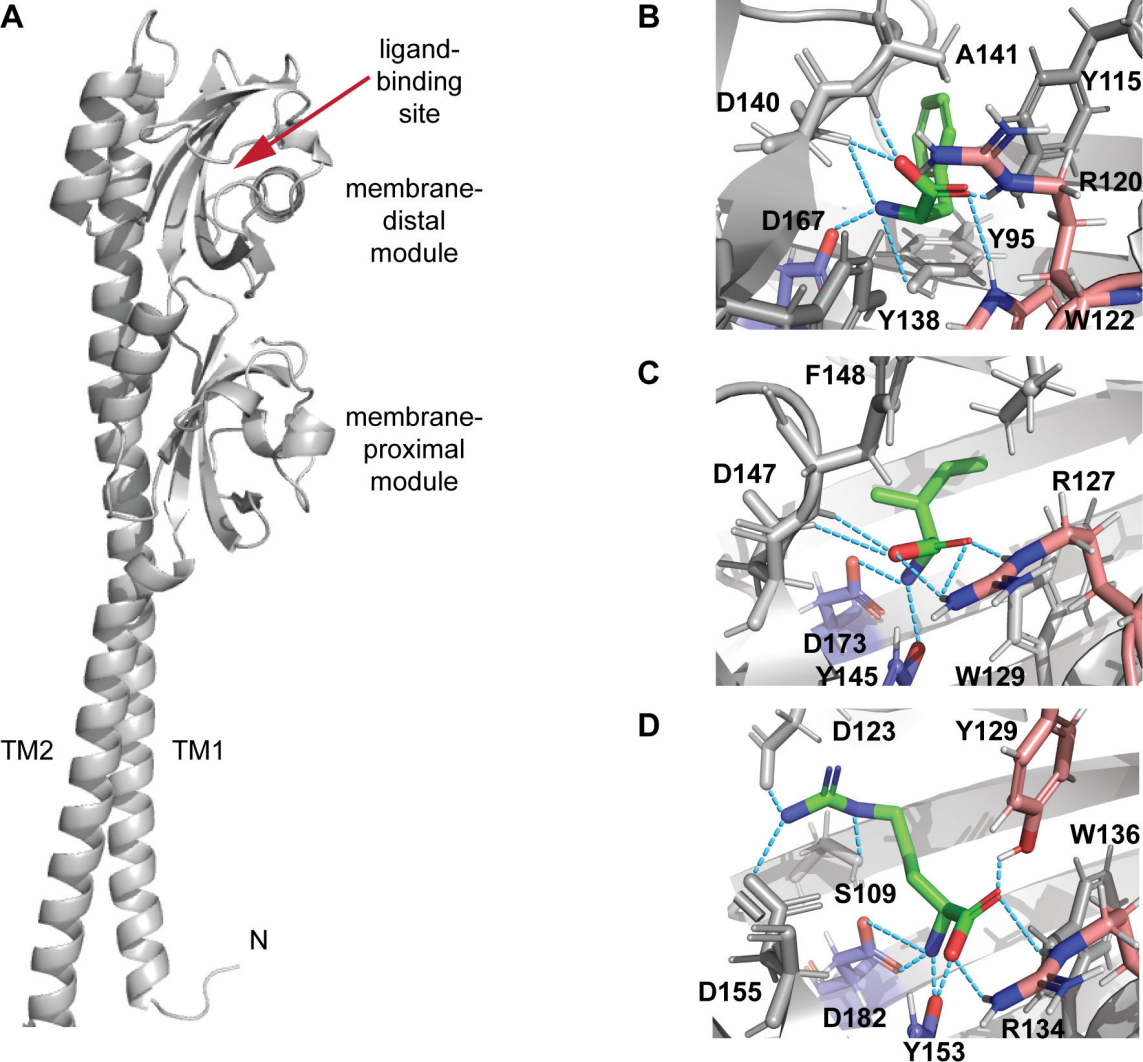
Computational docking of putative ligands to predicted dCache_1AA domains from human gut commensal bacteria. (**A**) The AlphaFold structural model of the dCache_1 domain and flanking transmembrane helices from *Enterobacter hormaechei* chemoreceptor (AA4). Docking of putative ligands to the distal module of selected predicted dCache_1 AA domains: (**B**) AA4 from *Enterobacter hormaechei* with L-Phe, (**C**) AA5 from *Agathobacter faecis* with L-Ile, and (**D**) AA6 from *Ligilactobacillus ruminis* with L-Arg. Amino acid residues highlighted in blue are making contacts with the amino group of the ligand, and amino acid residues highlighted in red are making contacts with the carboxyl group of the ligand. Structural models with docked ligands are available at https://zenodo.org/records/10806258.

As our goal was validation of the overall approach rather than comprehensive characterization of individual receptors and their exact ligand-binding repertoire, we selected only three out of six targets for further verification. Target AA4 was chosen because it showed the broadest specificity. Target AA6 was chosen because it showed the narrowest specificity, binding a single positively charged amino acid, L-Arg. Target AA5 was chosen because it showed a mid-range specificity with strong preference for hydrophobic ligands. Furthermore, AA4 and AA5 had relatively large thermal shifts compared to other targets. Several amino acid ligands that produced large increases in melting temperature for AA4, AA5, and AA6 in thermal shift assays (Fig. 3B) were chosen for further computational and experimental characterization. A similar rationale for selecting ligands was used to minimize the overall effort while providing sufficient evidence that the proposed framework works. In case of the broad-spectrum receptor AA4, we choose only a few ligands based on their properties. For example, thermal shifts produced by L-Arg and L-Lys were similar, and considering that both are positively charged, we have arbitrarily chosen only one of them, L-Lys, for further analysis. First, we built AlphaFold (69, 70) models of the selected targets and performed computational docking with one of the respective ligands for each of these sensory domains using DiffDock (71) (Fig. 4). The results showed that in all three cases, amino acid ligands (L-Phe for AA4, L-Ile for AA5, and L-Arg for AA6) were bound by their respective sensory domains exactly the same way, as seen previously in crystal structures (6) and AlphaFold models (7) for other dCache_1AA domains (Fig. 4). The first tyrosine residue of the conserved motif (Y115 in AA4), arginine residue, and tryptophan residue make contacts with the carboxyl group of the ligand, while the second tyrosine residue (Y138 in AA4) and aspartate residue make contacts with its amino group (Fig. 4B through D).

**Fig 5:**
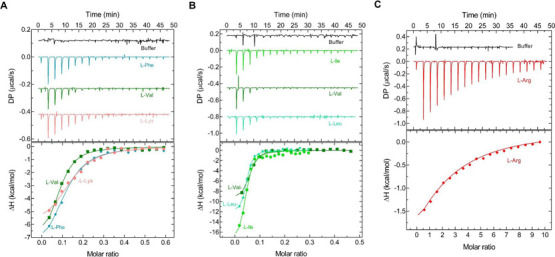
Microcalorimetric titration of selected recombinant dCache_1AA domains with amino acids. The upper panel upper shows raw titration data, and the lower panel shows integrated corrected peak areas of the titration data fit using the single-site model. Further experimental details are provided in Table S3. (**A**) AA4 from *Enterobacter hormaechei*, (**B**) AA5 from *Agathobacter faecis*, and (**C**) AA6 from *Ligilactobacillus ruminis.*

Experimental validation of ligand binding was then performed using *in vitro* isothermal titration calorimetry (ITC). These studies confirmed that AA4 is a broad-spectrum amino acid sensor, binding L-Phe, L-Met, and L-Lys (Fig. 5A), as well as eight other tested amino acids (Fig. S2), in agreement with the thermal shift measurements. Further validating the result of thermal shift experiments, we observed no binding to AA4 for L-Glu (Fig. S2). Binding of L-Ile, L-Leu, and L-Val to AA5 and binding of L-Arg to AA6 were also confirmed by the ITC studies (Fig. 5B and C).

### Verification of signaling initiated by ligand binding

**Fig 6:**
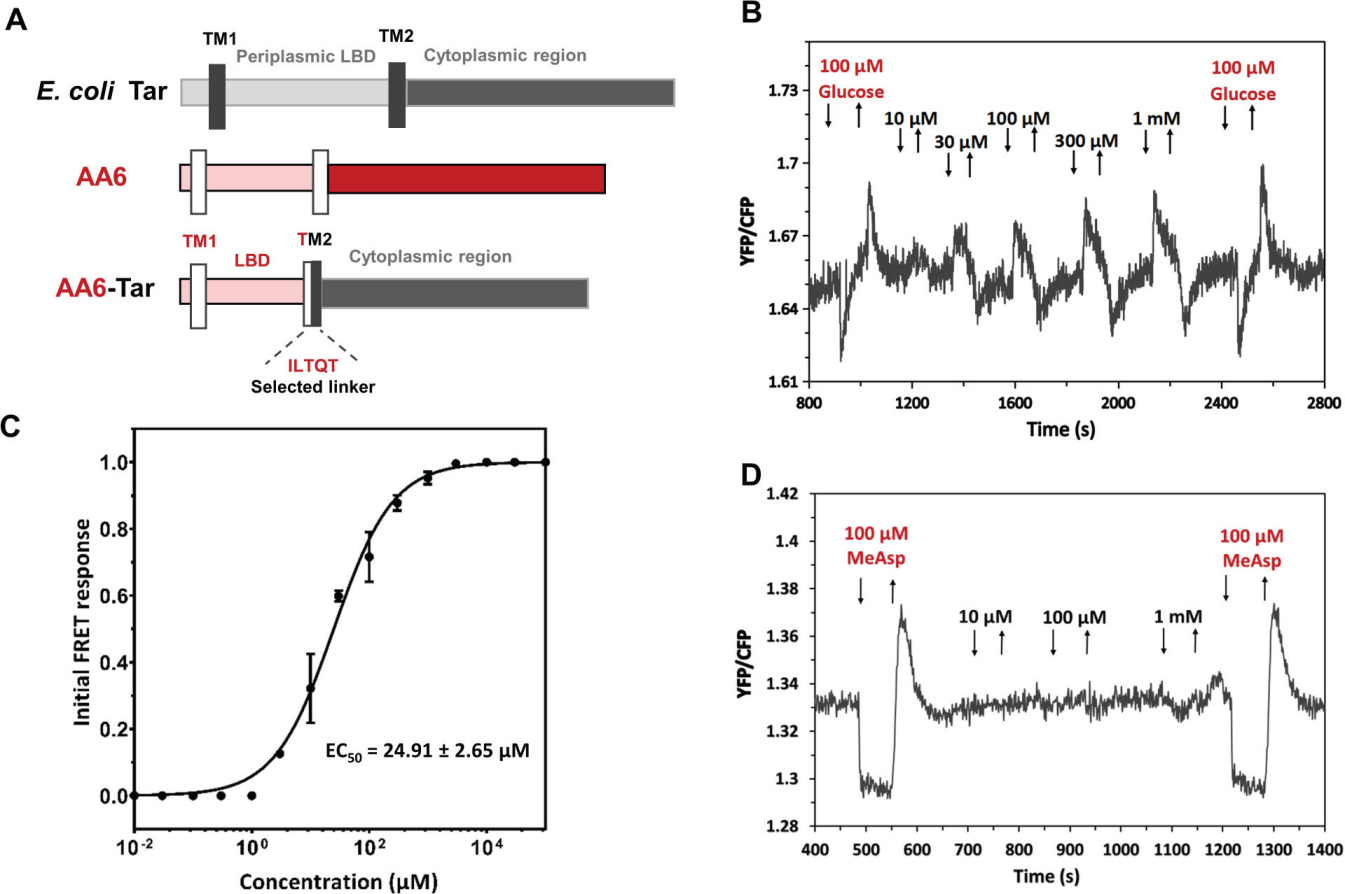
FRET measurements for *E. coli* cells expressing AA6-Tar hybrid. (**A**) Schematic representation of the hybrid receptor construction, with domains of tar shown in different shades of gray and domains of AA6 in different shades of red. Transmembrane (TM) sequences are indicated. The hybrid is composed of TM1 and ligand-binding domain of AA6 receptor, fused within TM2 to the cytoplasmic region of tar using the indicated linker. (**B**) FRET measurement of *E. coli* chemotaxis pathway response in cells expressing AA6-tar hybrid as a sole receptor to the indicated concentrations of L-Arg. Glucose was used as a positive control, since it elicits a chemotactic response in a receptor-unspecific manner. (**C**) Corresponding dose response mediated by AA6-tar hybrid with stimulation by L-Arg. Data were fitted using Hill equation, with the EC50 fit value being indicated. (**D**) FRET measurement of the wild-type tar, with the known ligand α-methyl-D,L-aspartate used as a positive control.

Ligand binding is necessary but not sufficient for signal transduction. In some cases, ligand binding to sensory domains does not elicit a cellular response (72), for example, due to insufficient conformational changes in the receptor molecule or binding site being different from that of specific effectors. To further verify whether ligands are capable of eliciting cellular responses, we constructed hybrid chemoreceptors for targets selected for in-depth validation, AA4, AA5, and AA6, by combining each sensory domain and the signaling domain of the *Escherichia coli* tar chemoreceptor. This approach enables testing signaling properties of a sensory domain in a heterologous *E. coli* system using a well-established assay based on Förster resonance energy transfer (FRET) (73, 74). However, only the AA6-tar hybrid (Fig. 6A) was functional. From the *in vivo* FRET measurements (Fig. 6B), we observed that this hybrid receptor could respond to L-Arg mediating a dose-dependent repellent-like response in the micromolar concentration range (Fig. 6B). The sensitivity of this *in vivo* response was higher (e.g., EC50 was lower [Fig. 6C]) compared to the *in vitro* binding affinity derived from ITC measurements (Fig. 5C). This is consistent with the previous FRET measurements of receptor responses and is likely explained by signal amplification by chemoreceptor clusters *in vivo* (74, 75). The repellent response observed in our FRET measurements may either indicate that AA6 indeed elicits a repellent response to L-Arg in its host or that the sign of the response has been inverted in the hybrid compared to the original receptor (76). No responses to L-Arg were observed for the wild-type *E. coli* tar (Fig. 6D). Thus, we conclude that AA6 binds L-Arg directly, and the ligand binding leads to signal transduction.

## DISCUSSION

The human gut is a habitat for thousands of microbial species whose activity has a profound impact on human health; however, most of these species remain unstudied, and their properties are poorly understood. Signal transduction is an important biological process that enables bacteria to sense changes in their microenvironment and adjust their cellular responses accordingly. To begin exploring the signals human gut commensals sense and respond to, we have developed a framework for the identification of sensory domains, the prediction of signals that they recognize, and their experimental validation.

To establish what types of sensory domains are common to human gut commensals, we compiled a list of these organisms from the literature and used the MiST database (77) to identify all transmembrane receptors encoded in their genomes. Sensory domains in these receptors were identified using sequence-to-profile and profile-to-profile searches. We identified 746 such domains (that belong to 15 different families) in 60 genomes of human gut commensals and found that most of them belong to the Cache superfamily, which was previously reported as the largest superfamily of extracytoplasmic sensory domains in bacteria and archaea (65). We then performed profile-to-sequence searches against the large UHGP catalog initiated with profile models for 15 domain families identified in the previous step, which resulted in the identification of ~17,000 sensory domains in the human gut commensal bacteria. This rich resource will provide ample opportunities for future exploration. Recent advances in defining specific motifs within sensory domains enabled the identification of cofactors and ligands across the large genomic landscape (8, 9, 65, 78). Here, we used one of these motifs (7) to identify more than 500 putative amino acid sensing domains in the UHGP catalog and experimentally verified several of them selected from different taxonomic groups and receptor types. Supporting the validity of our approach, thermal shift and ITC measurements demonstrated that all of these domains bind amino acids or their derivatives, but not other nitrogenous compounds, albeit with different amino acid specificities. Finally, we demonstrated that constructing chimera proteins, where a sensory domain with verified ligand-binding properties is fused with the *E. coli* chemoreceptor signaling domain (73), can be used as a final step of this framework to reveal whether a putative ligand not only binds to the sensory domain but also elicits signal transduction.

In addition to presenting a framework for exploring the sensory repertoire of the human gut microbiota, this study has offered several noteworthy observations. For example, a large-scale genomic survey of bacterial chemoreceptors revealed that their extracytoplasmic sensory domains mostly belong to two superfamilies—Cache and 4HB_MCP—in nearly equal proportions (3). However, our study showed that in gut bacteria, Cache domains significantly outnumber 4HB_MCP domains: this was observed in both genomic (Fig. 2A) and metagenomic (Fig. 2B) data sets. Whether this trend is observed across the bacterial domain of life has not been explored in full detail, although a previous genomic survey (65) and the number of entries for Cache versus 4HB_MCP domains in current public databases strongly suggest the Cache dominance. From this perspective, the apparent abundance of Cache domains in gut-inhabiting bacteria is not surprising and provides an excellent opportunity for identification of their ligands in the near future. In contrast to 4HB_MCP domains, where ligands can bind to different sites (79), Cache domains have a well-defined ligand-binding pocket (3), which can be confidently modeled by AlphaFold. Several ligand-binding motifs for Cache domains have been identified recently (7–9), which substantially improves prediction of the ligand class, as shown here for dCache_1 domains with a conserved amino acid binding motif. Furthermore, recent advances in computational docking, such as DiffDock (71), in combination with AlphaFold, offer new opportunities in ligand screening on a large scale.

The identification of hundreds of amino acid sensors in human gut bacteria highlights the importance of these molecules not only as nutrients but also as signals. Amino acids are abundantly present in the gut as the result of food breakdown and microbial metabolism (80). Indeed, many amino acids were shown to play an important role in the gut. For example, arginine has been shown to regulate the activity of the immune system (81) and increase diversity of the gut microbiota (82). Arginine is also a key metabolic regulator of the opportunistic pathogen *Eggerthella lenta*, suppressing its metabolic inactivation of the drug digoxin (83). Isoleucine and other branched-chain amino acids are metabolized by gut microbes to branched short-chain fatty acids such as 2-methylbutyrate (84); this microbial metabolism has been shown to reduce atherosclerotic progression in a mouse model (85). Aromatic amino acids such as phenylalanine are metabolized by some gut commensals, for example, *Clostridium sporogenes*, to produce certain metabolites (e.g., phenylpropionic acid) that can be detected in serum at high levels (86) and that are inversely associated with type 2 diabetes risk (87).

Although we have identified many amino acid sensors in gut bacteria, at this time, it is unknown whether amino acids are the largest class of small molecule signals in the gut. Arguably, more detailed and optimized experimental characterization will be required for in-depth characterization of individual sensors. Furthermore, sensors that recognize classes of signals other than amino acids still await elucidation; however, our results demonstrate that the framework presented here will aid in their identification. Finally, our general approach can also be used for identifying other types of domains in the human gut microbiota and for characterizing their biochemical properties.

## MATERIALS AND METHODS

### Compilation of the human gut commensal genomes

A list of 60 common commensals in the healthy human gut was curated based on literature review. Organisms were chosen based on how frequently they were mentioned in the relevant literature, their relative abundance (when available), and published data sets. Genomes for each organism were selected based on their BioSample data to confirm they were isolated from the human gut. After their isolation source was confirmed, genomes were chosen based on completeness. All genomes were from the NCBI RefSeq database (32).

### Sensory domain identification

The Microbial Signal Transduction Database (77) was used to search for common commensals from the gut with a preference for genomes with complete or chromosomal assembly. All known extracytoplasmic sensory domains present in histidine kinases, methyl-accepting chemotaxis proteins, second messenger turnover enzymes, and ser/thr protein kinases and phosphatases were included in our list of identified domains from MiST. For proteins where no sensory domain was detected by MiST between two transmembrane helices in the region >100 amino acid, this region was subjected to HHpred search (64) against PDB_mmCIF70_10_Jan and Pfam-A_v35 databases. The default parameters were used in HHpred with the exception of minimum cover of multiple sequence alignment (MSA) hits (%) changed to 10, and *E* value cutoff for MSA generation changed to 0.1.

Protein sequences from the Unified Human Gastrointestinal Protein catalog (30) were downloaded from the MGnify database (88) using the uhgp-95.tar.gz (human gut v2.0.1). Sensory domains were identified using HMMs from Pfam 35.0 (67) that are listed in Data Set S2 and the HMMER 3.2.1 suite (89).

### Multiple sequence alignment and motif identification

Local BLAST (90), v.2.8.1+, was performed using UHGP-95 clusters (i.e., sequences clustered at 95% amino acid identity) as the database, and YP_003612116.1 from *Enterobacter cloacae* as the target amino acid sensor (7) sequence. Alignments were built using MAFFT v.7 with L-INS-I iterative refinement method (91) and were viewed using Jalview 2.11.2.6 (92) to verify the completeness of the amino acid binding motif—[YFWL]….[RK].W[WYF]{n1}[YF]{n2}[D] (7).

### Structure modeling and computational docking

Structural models were built using AlphaFold v.2.1.0 (69, 70) on a high-performance computing cluster. Canonical amino acids were downloaded using the Zinc20 Database (93) in the SDF format. DiffDock (71) was used to computationally dock canonical amino acids to dCache_1 domain models containing the amino acid binding motif. Docking results were viewed and interpreted using Pymol v.2.4.1 (94).

### Bacterial strains, plasmids, and culture conditions

Bacterial strains and plasmids are listed in Table S1. For chemotaxis and FRET experiments, *E. coli* was grown in TB medium (1% tryptone and 0.5% NaCl) at 34°C. For molecular cloning and protein expression, *E. coli* was grown in Luria-Bertani (LB) medium at 37°C. When necessary, antibiotics were used at the following final concentrations: kanamycin, 50 µg/mL; ampicillin, 100 µg/mL; and chloramphenicol, 34 µg/mL.

### Cloning, expression, and purification of dCache_1AA domains

The DNA fragments encoding the studied sensory domains were synthesized and cloned into pUC57-Kan cloning vectors by GenScript (https://www.genscript.com/). To overexpress these proteins, the periplasmic ligand binding domains between two transmembrane domains were amplified by PCR reaction, and the DNA sequences are listed in Table S2. The resulting fragments containing the overlapping part of pET28b(+) vector were cloned into the linearized expression vector pET28b(+) digested with NdeI and BamHI using Gibson assembly reaction in NEBuilder HiFi DNA Assembly Master Mix (New England BioLabs). *E. coli* BL21 (DE3) harboring the LBD expression plasmid was grown in 5-L Erlenmeyer flasks containing 1-L LB medium supplemented with kanamycin under continuous stirring (200 rpm) at 37°C. When OD_600_ reached 0.6, 0.1-mM isopropyl-β-D-thiogalactoside (IPTG) was added to induce protein expression. Growth was continued at 16°C for 12 h, and cells were harvested by centrifugation at 10,000 × *g* for 30 min at 4°C. Proteins were purified by metal affinity chromatography as described previously (73). Briefly, cell pellets were resuspended in binding buffer (20 mM sodium phosphate, 500 mM NaCl, and 20 mM imidazole, pH 7.4) supplemented with 0.2-µg/mL lysozyme, 1 mM MgCl_2_, and 1 mM PMSF, stirred for 30 min at 4°C and lysed using a sonicator. After centrifugation at 20,000 × *g* for 30 min, the supernatant was loaded into His GraviTrap column pre-equilibrated with binding buffer, and target proteins were eluted by elution buffer (20 mM sodium phosphate, 500 mM NaCl, and 500 mM imidazole, pH 7.4). The eluted proteins were dialyzed with the dialysis buffer (10 mM sodium phosphate, 150 mM NaCl 10% (vol/vol) glycerol, pH 7) and concentrated using Amicon Ultra-15 centrifugal filters.

### Thermal shift assays

Thermal shift assays were performed in 384 microtiter plates using a Bio-Rad CFX384 Touch Real-Time PCR instrument with the commercially available compound array_Biolog PM3B (https://www.biolog.com/wp-content/uploads/2023/12/00A-042-Rev-D-Phenotype-MicroArrays-1-10-Plate-Maps.pdf), as described previously (73). The compounds in Biolog PM3B were dissolved by adding 50-µL Milli-Q H_2_O to a final concentration of approximately 10–20 mM. Each 25-µL assay mixture contained 20.5-µL purified protein (30–100 μM) in phosphate buffer (10 mM sodium phosphate, pH 5.5), 2 µL SYPRO orange (Invitrogen) at 5× concentration, and 2.5 µL compounds from the Biolog PM3B. Samples were heated from 23°C to 95°C at a scan rate of 1°C/min. The protein unfolding curves were monitored by detecting changes in fluorescence intensity. The resulting data permitted the calculation of the midpoint of the protein unfolding transition or melting temperatures using the first derivative values from the raw fluorescence data, which was analyzed by the Bio-Rad CFX manager v.3.1 software.

### Isothermal titration calorimetry

Ligands and proteins (Table S3) were diluted with a buffer containing 10 mM sodium phosphates; 150 mM NaCl; 10% glycerol; pH 7.0. The purified proteins were titrated in the sample cell at a final concentration of 50–65 µM each. The protein concentrations were predetermined by absorbance at 280 nm. The ligands were placed in the titration syringe at a nominal concentration of 0.1 to 2.5 mM (Table S3) to saturate the protein sample during the titrations. All the measurements were performed at 25°C with the instrument MicroCal PEAQ-ITC (Malvern Panalytical) with a method consisting of 19 injections (first, 0.4 µL, and the rest 2 µL each) and 150 s of spacing. The raw data were processed with the MicroCal PEAQ-ITC Analysis Software (Malvern Panalytical) using the “one set of sites” models and plotted using GraphPad Prism (GraphPad Prism Corp., San Diego, CA, USA). Detailed experimental conditions are listed in Table S3.

### Construction of hybrid chemoreceptors

For the constructions of hybrid receptors, the coding sequences of AA6-Tar hybrid were amplified using PCR (oligonucleotide sequences are shown in Table S1). The amplified fragments containing overlapping sequences of vector pKG116 were ligated into the digested vector pKG116 using the Gibson assembly reaction. After cloning, the functional hybrid receptors were selected from a library of AA6[1–X]-XXXXX-Tar[203–553] as described previously (74). Briefly, the library was applied to a soft agar plate with glucose gradients for three rounds of selection. Cells with the best attractant responses to glucose were applied to the edge, accumulating at the plate’s center. After three rounds of selection, most chemotactic cells were streaked out on LB plates to obtain single colonies. The sequence of the linker in the functional hybrid was identified by DNA sequencing.

### FRET measurements

FRET measurements were performed as described previously (95, 96). Cultures of the receptorless *E. coli* strain VS181 expressing AA6-Tar hybrid (or wild-type Tar) and CheY-YFP/CheZ-CFP FRET pair were prepared by inoculating 200 µL of the overnight culture into 10-mL tryptone broth (TB) medium supplemented with appropriate antibiotics and inducers (50 µM IPTG and 1 to 2 µM sodium salicylate) and grown in a rotary shaker at 34°C and 275 rpm. Cells were harvested at an OD_600_ of 0.5 by centrifugation and were washed twice with tethering buffer (10 mM KH_2_PO_4_/K_2_HPO_4_, 0.1 mM EDTA, 1 µM methionine, 10 mM sodium lactate, pH 7.0). For microscopy, the cells were attached to the poly-lysine-coated coverslips for 10 min and mounted into a flow chamber that was maintained under constant flow of 0.3 mL/min of tethering buffer using a syringe pump (Harvard Apparatus) that was also used to add or remove compounds of interest. FRET measurements were performed on an upright fluorescence microscope (Zeiss AxioImager.Z1) equipped with photon counters (Hamamatsu). The fluorescence signals were recorded and analyzed as described previously (76).

## Data Availability

Multiple sequence alignment and structural models are available at https://zenodo.org/records/10806258.

## SUPPLEMENTAL MATERIAL

Data Set S1 mbio.01039-24-s0001.xlsx Common human gut commensal bacteria.

Data Set S2 mbio.01039-24-s0002.xlsx Extracytoplasmic domains identified in genomic data.

Data Set S3 mbio.01039-24-s0003.xlsx Extracytoplasmic domains identified in metagenomic data.

Data Set S4 mbio.01039-24-s0004.xlsx dCache_1AA domains identified in genomic and metagenomic data.

Data Set S5 mbio.01039-24-s0005.xlsx dCache_1AA domains selected for experimental validation.

Supplemental material mbio.01039-24-s0006.pdf Supplemental figures and tables.

ASM does not own the copyrights to Supplemental Material that may be linked to, or accessed through, an article. The authors have granted ASM a non-exclusive, world-wide license to publish the Supplemental Material files. Please contact the corresponding author directly for reuse.
