# Supplementary material for: Framework for exploring the sensory repertoire of the human gut microbiota
Source: mBio. 2024 May 17;15(6):e01039-24. doi: 10.1128/mbio.01039-24 (PMC11237719; doi:10.1128/mbio.01039-24)
Supplement: Supplemental material — Supplemental figures and tables. [file mbio.01039-24-s0006.pdf]

Supplementary Figures and Tables

Framework for exploring sensory repertoire of the human gut microbiota

Patricia A. Ross[1,2#], Wenhao Xu[3,4#], Ekaterina Jalomo-Khayrova[3,4,5], Gert Bange[3,4,5], Vadim M. Gumerov[1,2], Patrick H. Bradley[1,6], Victor Sourjik[3,4*], Igor B. Zhulin[1,2*]

[1]Department of Microbiology, The Ohio State University, Columbus, OH, 43210 USA

[2]Translational Data Analytics Institute, The Ohio State University, Columbus, OH, 43210 USA

[3]Max Planck Institute for Terrestrial Microbiology, Marburg, 35043, Germany

[4]LOEWE Center for Synthetic Microbiology (SYNMIKRO), Marburg, 35043, Germany

[5]Department of Chemistry, Philipps-University Marburg, Marburg, 35043, Germany

[6]Infectious Diseases Institute, The Ohio State University, Columbus, OH, 43210 USA

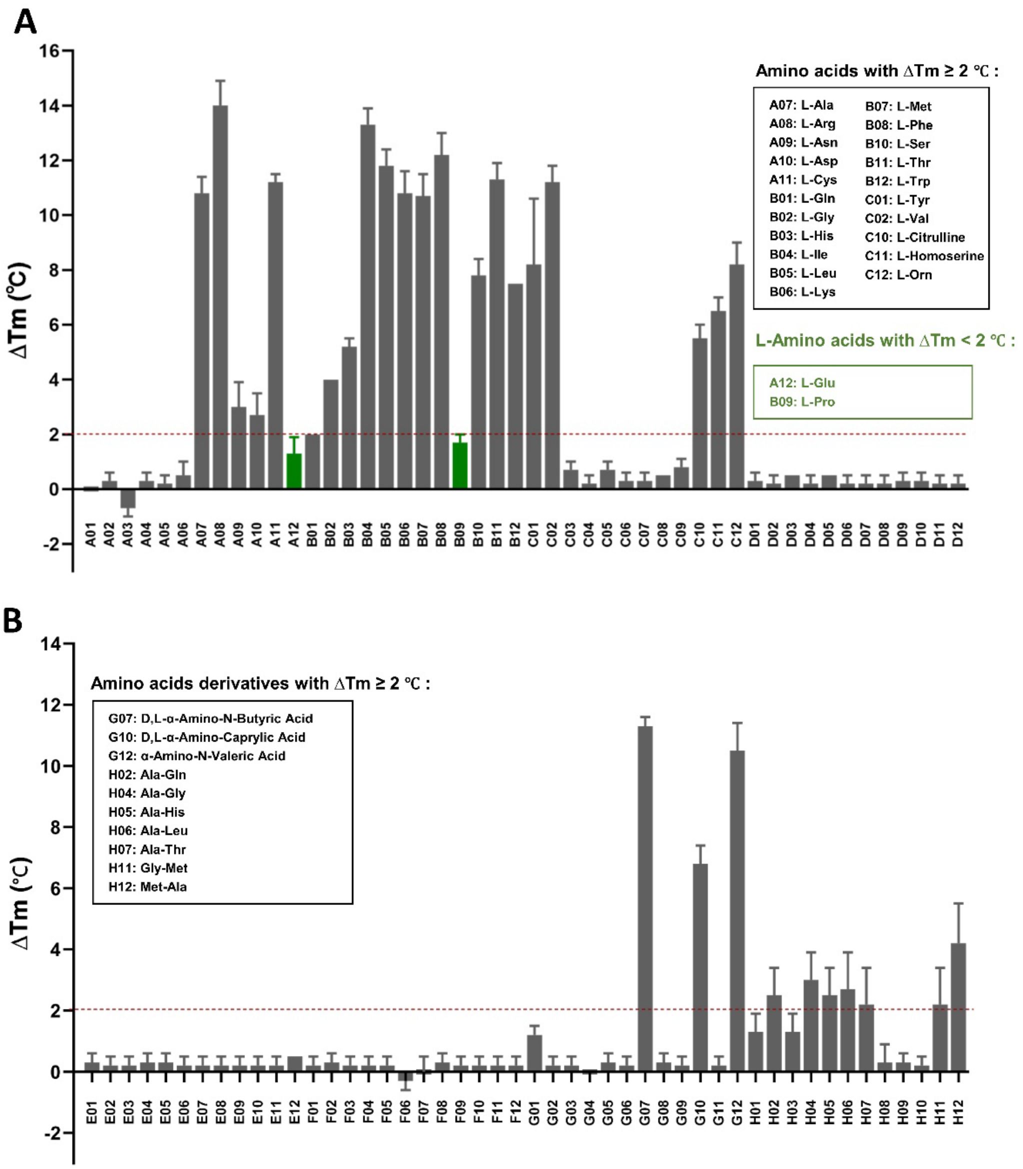

Fig. S1. Thermal shift assays-based ligand screening of AA4. Shown are changes in the protein melting temperature (Tm) induced by the indicated compounds present in Biolog PM3B compound array of nitrogen sources which were separated into (A) the first half and (B) the second half. The detailed layout of Biolog PM3B can be found using this link: https://www.biolog.com/wp-content/uploads/2023/12/00A-042-Rev-D-Phenotype-MicroArrays-1-10-Plate-Maps.pdf . The red dashed line indicates the threshold of 2°C used as a significance cutoff for ligand identification. The numbers below the bar represent the location of each amino acid in the Biolog PM3B plate. Amino acids that elicited thermal shift of ⩾ 2°C (labeled in

black) were considered positive; compounds with ΔTm < 2°C were considered negative, particularly two L-amino acids (labeled in green). Data are shown as the means and standard deviations of results from three biological replicates, each conducted in triplicate.

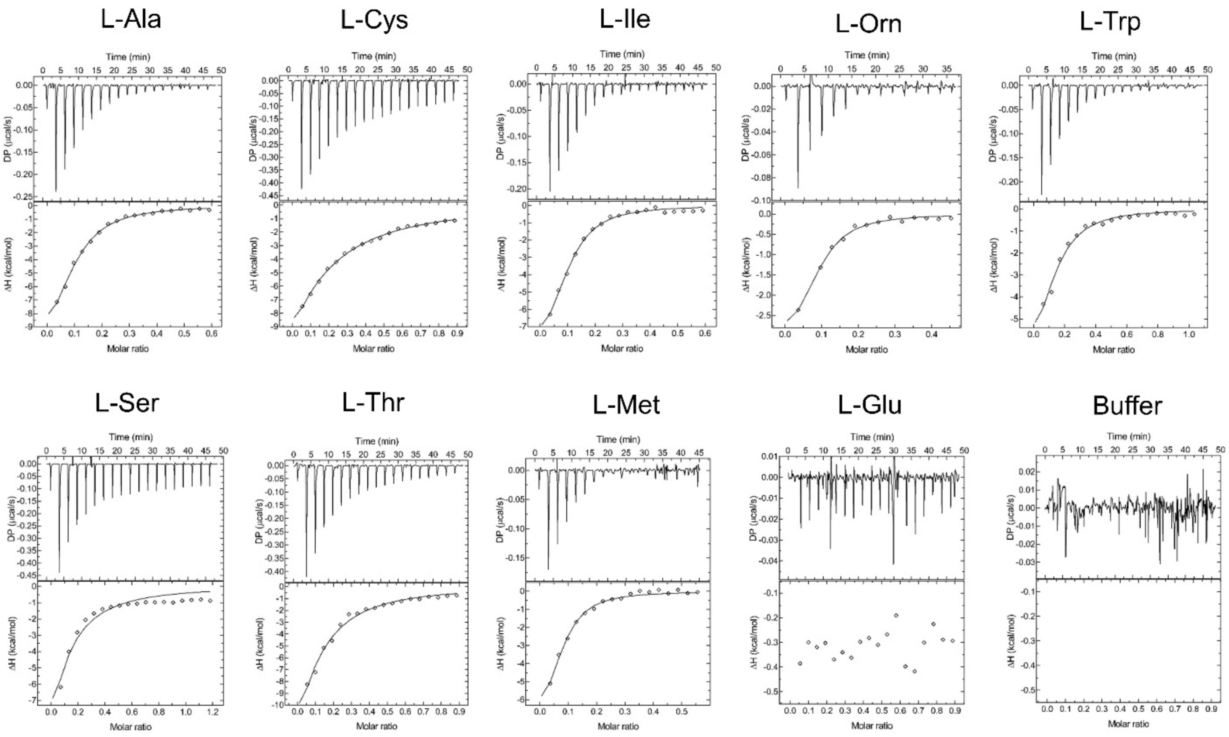

Figure S2. Confirmation of ligand binding to AA4 using isothermal titration calorimetry. ITC measurements for eight amino acids that were identified as putative positive ligands from thermal shift assays (ΔTm ⩾ 2℃), and for L-Glu that showed a thermal shift of less than 2℃. Upper panels: raw titration data. Lower panels: integrated, dilution heat corrected and concentration-normalized peak areas of the titration data fitted the single-site binding model. The related experimental conditions and binding affinities are shown in the Table S4.

Table. S1. Strains, plasmids and oligonucleotides used in this study.

Strains and plasmids	Genotype or relevant characteristics[a]	Reference
**Strains**		
Escherichia coli BL21(DE3)	F^- *ompT gal dcm lon hsdS*$_B$($r_B^- m_B^-$) λ (DE3 [*lacI lacUV5-T7p07 ind1 sam7 nin5*]) [*malB*$^+$]$_{K-12}$($λ^S$)	(1)
E. coli DH5α	F^- *endA1 glnV44 thi-1 recA1 relA1 gyrA96 deoR nupG purB20* φ80d*lacZ*ΔM15 Δ(*lacZYA-argF*) U169, hsdR17($r_K^- m_K^+$), $λ^-$	(2)
E. coli UU1250	Derivative of RP437; Δ*aer*Δ*tsr*Δ(*tar-tap*) Δ*trg*	(3)
E. coli VS181	Derivative of RP437; Δ(*cheYcheZ*)Δ*aer*Δ*tsr*Δ(*tar-tap*) Δ*trg*	(4)
**Plasmids**		
pET28a (+)	Km^R; Protein expression vector	Novagen
pSB13	Cm^R; Tar expression plasmid, pKG116 derivative, T768 was mutated to C768 to remove the NdeI restriction site	(5)
pKG116	Cm^R; Expression vector, salicylate inducible; for generation of hybrid chemoreceptor	(6)
pVS88	Ap^R; CheY-EYFP / CheZ-ECFP expression plasmid used for FRET measurements	(4)
pET28_ MGYG000001518 -01147 _LBD	Km^R; pET28a (+) derivative containing a DNA fragment encoding MGYG000001518-01147_LBD	This study
pET28_ MGYG000001473 -00406 _LBD	Km^R; pET28a (+) derivative containing a DNA fragment encoding MGYG000001473-00406 _LBD	This study
pET28_ MGYG000017777 -02370 _LBD	Km^R; pET28a (+) derivative containing a DNA fragment encoding MGYG000017777-02370 _LBD	This study
pET28_ MGYG000063141 -00874_LBD	Km^R; pET28a (+) derivative containing a DNA fragment encoding MGYG000063141-00874 -LBD	This study

pET28_ MGYG000246596 -00160_LBD	Km^R; pET28a (+) derivative containing a DNA fragment encoding MGYG000246596-00160 -LBD	This study
pET28_ MGYG000151212 -00948_LBD	Km^R; pET28a (+) derivative containing a DNA fragment encoding MGYG000151212-00948 -LBD	This study
pET28_ MGYG000210351 -01325_LBD	Km^R; pET28a (+) derivative containing a DNA fragment encoding MGYG000210351-01325 -LBD	This study
MGYG000210351 -01325-Tar	Cm^R; pKG116 derivative containing a DNA fragment encoding MGYG000210351-01325 [1-298]-ILTQT-Tar [203-553];	This study

[a]Ap, ampicillin; Km, kanamycin; Cm, chloramphenicol

**Oligonucleotides**	**Sequence (5'-3')**	**Purpose**
MGYG000001518 -01147-LBD_F	GTGCCGCGCGGCAGCCATATGCGTTCAGCGAAAACGAGT	Construction of pET28_ MGYG000001518 -01147-LBD
MGYG000001518 -01147 -LBD_R	ACGGAGCTCGAATTCGGATCCCTAGGTAGTTTTGATAATA GGGAA	
MGYG000001473 -00406 -LBD_F	GTGCCGCGCGGCAGCCATATGAACTACCGTACGTCATCC	Construction of pET28_ MGYG000001473 -00406 -LBD
MGYG000001473 -00406 -LBD_R	ACGGAGCTCGAATTCGGATCCCTAAGAACGTGTGACCAT CGA	
MGYG000017777 -02370-LBD_F	GTGCCGCGCGGCAGCCATATGCAGAGTCGTAAAGCAGC	Construction of pET28_ MGYG000017777 -02370-LBD
MGYG000017777 -02370-LBD_R	ACGGAGCTCGAATTCGGATCCCTAGTTGCGGACGGCTTTC	
MGYG000063141 -00874-LBD_F	GTGCCGCGCGGCAGCCATATGAATTTCCAGGTCACGCGC	Construction of pET28_ MGYG000063141 -00874-LBD
MGYG000063141 -00874-LBD_R	ACGGAGCTCGAATTCGGATCCCTAGCTGGCTTTCAGCAAT GAA	
MGYG000151212 -00948-LBD_F	GTGCCGCGCGGCAGCCATATGACCAAAAATACCATGGTA CA	Construction of pET28_ MGYG000151212 -00948-LBD
MGYG000151212 -00948-LBD_R	ACGGAGCTCGAATTCGGATCCCTAGTTACGCAGAATGGA TTG	
MGYG000210351 -01325-LBD_F	GTGCCGCGCGGCAGCCATATGGAGCGGAACAATCTGAGT	Construction of pET28_ MGYG000210351

MGYG000210351 -01325-LBD_R	ACGGAGCTCGAATTCGGATCCCTAATGTCTCTCATTGTTA AGGTC	-01325-LBD
pKG116-seq_F	AAGCCATAAGGAGTACCATATG	Sequencing for hybrid Chemoreceptor
pKG116-seq_R	TTACTTATTTATCCGCGGATC	
pET28a-seq_F	GTGCCGCGCGGCAGCCATATG	Sequencing for LBD expression plasmids
pET28a-seq_R	ACGGAGCTCGAATTCGGATCCCTA	
MGYG000210351 -01325-Tar _F1	AGCCATAAGGAGTACCATATGATGAATAACAACACAAGC TAC	Construction of MGYG000210351 -01325-Tar hybrid chemoreceptor
MGYG000210351 -01325-Tar_R1	CCACCAGCAGAATCAANNNNNNNNNNNNNNNGACCGCA ATGACCCCC	
MGYG000210351 -01325-Tar_F2	TTGATTCTGCTGGTGGCGTG	
pKG116-Tar_R2	TACTTATTTATCCGCGGATCCTCAAAATGTTTCCCAGTTT GGATCTTG	

References

1. Jeong H, Barbe V, Lee CH, Vallenet D, Yu DS, Choi SH, Couloux A, Lee SW, Yoon SH, Cattolico L, Hur CG, Park HS, Ségurens B, Kim SC, Oh TK, Lenski RE, Studier FW, Daegelen P, Kim JF. 2009. Genome sequences of Escherichia coli B strains REL606 and BL21(DE3). J Mol Biol 394:644–652.

2. Woodcock DM, Crowther PJ, Doherty J, Jefferson S, Decruz E, Noyer-Weidner M, Smith SS, Michael MZ, Graham MW. 1989. Quantitative evaluation of Escherichia coli host strains for tolerance to cytosine methylation in plasmid and phage recombinants. Nucleic Acids Res 17:3469–3478.

3. Ames P, Studdert CA, Reiser RH, Parkinson JS. 2002. Collaborative signaling by mixed chemoreceptor teams in Escherichia coli. Proc Natl Acad Sci U S A 99:7060–7065.

4. Sourjik V, Berg HC. 2004. Functional interactions between receptors in bacterial chemotaxis. Nature 428:437–441.

5. Bi S, Jin F, Sourjik V. 2018. Inverted signaling by bacterial chemotaxis receptors. Nat Commun 9.

6. Burón-Barral MDC, Gosink KK, Parkinson JS. 2006. Loss- and gain-of-function mutations in the F1-HAMP region of the Escherichia coli aerotaxis transducer Aer. J Bacteriol 188:3477–3486.

Table. S2. The synthesized DNA fragments encoding individual dCache_1AA domains .

ORF<br>(periplasmic sensory domain of the protein)	DNA sequence (5'-3')
AA1<br>MGYG000001518-01147<br>(1-888)	CGTTCAGCGAAAACGAGTCTGCATAATGAGGTCCAAAAAACTATTGATGAAAATGTGGCAATGCTGAACTCGGTGCTGAACGACGCCAT<br>TGATCAAAAGCTGAAACAGGTTTCAACGATGTCTGCGCTGTTTAATAAATCGGAAAAACCGGCAGAAATAACAAAATCTCTTTTTAGCT<br>ACGCGTCCCTGCACCCTGAGGTTGCCTCCGTCTACGTAGGAACTGAGCAGGGCGGTATGATCCTTGCGCCGAGCGCCACCCTACCAAAA<br>GACTTTGATCCACGCACCCGTCCGTGGTATAAAGATGCCGTTGCCGCGAAAGGGAAGGTTGTGATCACAAGCCCCTACAAAACGGCGGA<br>TGGAACCGATGCTATGGTGGTGACCATTGCGCAAACTTTGAGCGATAACTCGGGGGTGGTGGGTATTGACTTAAGTTTAGACCGCATCA<br>CCAGCATAGCACGTACCGTCAGTTTCGGTAAGAAAGGCTATGCGTTCATTCTGGACCAGGAACGGCATGTTATCACCCACCCGAGCTTA<br>GAAAATGGCTCCGAAGTCGCTGGCAAAGACATGGACAGCCTGTTTGCCGATGATGCAGGCCGCACGAAAATTGGCAAAGGCGATGAAA<br>AAGTTATGGTAGCGTTCGTGACCAACGAATTGACAGGTTGGAAGCTGGCAGGTGCCTATCCGCAGAAGGAAATCGATGAGGCTGTCTTC<br>CCTATTATCAAAACTACC
AA2<br>MGYG000001473-00406 (79-954)	AACTACCGTACGTCATCCCGGCACATTATTGATGGCCAACTGGGAAGCCTGGAAGAGGCCGGCAATCGCTACGGGAAGGAAATCGACG<br>GATGGCTGCGCGTTCAGGGTAAAATCATCGAGGAGATGGAACAAGATCTGAAATATCAGGATAACCAGAAAGCGGATAAAAAGGATGA<br>TGAAAAGAATGAAAAAGATGAAAAAGACGAAAAAGATGAAAAGGATAAAAAAGACAACATATATGGGCCTGAATATTTGAGCAAATA<br>CTTTAAATATAAAAACGACACCAATCCAGACATCATTGAATACTTCATGGGTCTGCCAGAGGAAAACAAACTGGTAACCCACTTAGGCT<br>ACATTCCGATCCCGGATGATTATAACGTGCGCGAAAAAGATTGGTATATTCGTGCAGACGAATCAAATGGCGTGGCCCTGAGTAAACCG<br>TATGTGGATGTCGAACATGAGAAAGTGGTGGTGACCATGGGCAAAGCGATTAAGGAAAACGGTAAAACGAAGGCTGTACTTTGCTCTG<br>ACATTTTCATCGATCATCTTATGGACATTACCAGCAAAGCGAAACCGTTGAAAGGCTCCAAAGGTTTCCTGATTGACGACGATGGGAAC<br>ATCCTCGTTCATTATAACAAAGATTTTATGTACTCCAAAGAAAAAGGTTTTACTAAAGCAACTGATGTGATGGGCAAGGAGATTGACAA<br>ACTGATAAAAAATAAAGATGGCAAAAAAAACATCGAAATTATTAAAGATTATGATAATGTGGACAAATATTTTCTGACGGTTCCCATCG<br>AATACAGTAATTGGACCATTGGTTTCTCGATCCCTGTTAAGGAGGTTGAGCAGCCGTTAAATTCGATGGTCACACGTTCT
AA3<br>MGYG000017777-02370<br>(85-891)	CAGAGTCGTAAAGCAGCCGTTGAAGATTTTTCTGCGAATGCGGGTGGCCGGCTCCGCTTGGCGGGGCAGTATTTGTCCGAAATGATCGG<br>CAACTCCAAAGGTATTGTTGACATCTTAGCTCAGCTGCCTAGCGTTGCGGATGCAGATGGTCAGGTTACCTCGTATGCGGCAAATACAG<br>AAAGCGTAAAACCCCTAGGCGCAGAACTGAGTGGCCCGGAACGTGTATTGTATGATGAGTTCAGCACATTTACCAAGGTGTACCCGCAG<br>TTCGAGTTAATCTACTACGGGACGAGAGATGGAGGCTTTGTTCAAGCACCGGATGATACGCTGTCCGCCGGCTTCGATCCGCGAAAACG<br>CCCATGGTATGAAGCGGCGCAAAAATCGGATGGCGTCGTCTTAACCGAATTTTATCAGTCAGATAACGGTAACCTTGTGACCACTGTGG<br>CGAAATCTGTGCGTCCAGACGGCAAACTGCTGGGTGTGGTGGGGATTGACATCAATTTAAACGCGCTGAGCACCTTCCTGGACACCCTC<br>AAGTTGGGTAAGACAGCGCGTCTGCTGCTGACCGAAGATACTGGACTGATTTTATATAACCCGACCCGTCCTGAGCGCGTGCTGAAAAA<br>CATTCGCGACGTTGGCGAACCGGGGCTGGTGGCTGCCTTTGATCAAGGCACTGGTAGCGGTGAAGTGTCTTACGATGGTAAAACCTTTG<br>TGGCCGTCAGTCAAGTAACGCAGGGAGGTTGGCATCTGATCGCACTCGTTGACCGTGCCGAGCTTCTGGACAATGCCATGAAAGCCGTC<br>CGCAAC
AA4<br>MGYG000063141-00874<br>(82-813)	AATTTCCAGGTCACGCGCCAGGACAACCAGCAGTCCCAGCGTGATATCCTGACGAGTACCTCCGCGAGTCATAACATGGCGATCGCTGA<br>CTGGGTGAAATCTAAAATGACTGTGATCGCCTCGGCCCAGAGTATTGCACTGGCAGATGACCCGGTGCCGGTGTTTAAACAATTGGCGC<br>AGGCCGGTGGCTTTACTAATGTCTACGCGGGCTATGCGTCAAAAACTGCTAAGTTTTCTGATCCGGCAGGTGTTCCAGCGGATTACGATC<br>CTACCATTCGTCCCTGGTATCAACAAGTCGTTTCCACGGACGGACCGGTAGTCACGGCGCCGTATGTGGATGCGGGGACGGGCAAGCTG<br>GTGGTTACCTTTGCTGTGCCGGTGAAAGAAAATGGAACCCTGAAGGCAGTCGTGGCAGGTGATGTTGCCATGGATTCCGTTGTAGCGAA<br>CGTGCGGGGCATTCACCCAACCCCTGCTAGCAGCGGTTTACTGCTTAACAGCGACGGTACTGTAATTGCCGCAAACGAACCGACCCTGA<br>CGTTAAAACCATTTACAGGCACCATTAAAGGGATTGATTTAGCGGCACTGAAATCGGGCAACCCGGTTGATGGTACCCTGAACGATGAA<br>GAGAAAACCTTCATCGCTTCGGCCGTCCCCGGCACCAATTGGCTGCTCGTCGTGGCGCTGGACTCAAGTGACGCAACGAGCGGGATGCG<br>TTCATTGCTGAAAGCCAGC
AA5<br>MGYG000151212-00948	ACCAAAAATACCATGGTACAGAATGAAAAGCAGTTGCTGACGGCCAACGCAGATACGAATGCGCAGGTCATCGATCAGTGGCTGTTGA<br>AAGAAGCGGGGATCGTCCACACTATGCGCGATACCCTGGCTTATATGAATACCAAAGATGGCGAAACCGTCATGAATTATCTGGAGAAG<br>CAGCTGGGTGAGAATGAGGATGCCCTAATGTATTACTGCTGTTTGGAGAAAGAAAAAAGCGTTTTACCGGCAGATCATAGTACCTTAGA

(97-822)	TCTGGACCCGACTGAGCGTGACTGGTGGAAACAAGCTGTTTCAGAAAACGGGCTTATTTTTACCGCGCCTTATACGGACTTTGTCACCGG<br>CCAAATGATTGTGACAATTGCAGAACCGCTGACACTCGAAGGCGATACGGCCGTGATACTCGCGGACATCACCATCGATAAGCTGGTGG<br>AAATGACGCAGCAGATCCGCGATTCTAAAACCGAAACGTTCCTGCTCGCGAGCGACGGTTCCGTAATTACACATGAAAACAAGGAATAC<br>CTACCAAAAGAAGAGGGTAACACCGTTCTTACGGATAAAGTGAACATCAAACTGGATAGCAAAACTACGACGGCGTTTCGCGATTACG<br>ACGGCCAGAACAAATATTTAGCTATCGGAAACGTTGACAAAACCGGCTGGAAACTGGGTGTGATGCAAAAGACTTCGGTGATTCGTCAA<br>TCCATTCTGCGTAAC
AA6<br>MGYG000210351-01325<br>(136-861)	GAGCGGAACAATCTGAGTAAGGAATCAGCCGTTGATCTGATTCAGGAAGAACGCGAGCACTTGCAAAAGACTTCGGAAGCAATGCTGA<br>AAAAAATGGCAACCTATCCGGAATTTACCGCCAAAAAATTCGACCGCCAGCAGCTGGCTCGTGAGGTCAAAAAAACCTGCAACGCGAA<br>CGACGCGTTACTGTCGACAACTGTAGCTGATCAGGATGATAACTATGCGTCGTCCGATAAGCTCCCAGCAGGCTACAAACCTACCAGTC<br>GTGAGTGGTATCAGAAAGCGGTTGCGGACATGGGTAACGTTATTTGGACCCTTCCGTATAAGGATGCGGAAAGCGGCGTCTACGTGATT<br>ACGGCCGCCTATGCATACAAAGCGCAAGATGGTAAAACACGAGTGGTGACTAGCGATGTCTCCTTTAATGCCCTGGAACGCCCGCTGGA<br>CAACCTGCGTATCGGTCACACCGGACGTGTGACGCTGGTGTCCGACGATGGCATCGCCTTGGCATCTAAAGGCGCTATCGATGATAGCC<br>ATTATAGCCGCGGCAATAATCTGAGTAATGATAAAATGTACAAAGCGATTAAAGCAAGCAACAAGCGCAAAGGCTTTGTACATATCGA<br>CGGTGAAAGCAAGGTGGCTGATGTATACTTCAATAAAGGTGAAGCCGGTTCTGAATATTGGGCGTATGCCTACGTTATGGCGAGTGACC<br>TTAACAATGAGAGACAT

Table S3. Microcalorimetric studies of selected dCache_1AA domains from human gut bacteria. For each protein its organism, identifier, affinities, experimental conditions and enthalpy changes induced by the addition of relevant ligands were shown.

Protein			Ligand		K_D (μM)	K_D Error (μM)	Δ*H* (kcal/mol)	Δ*H* Error (kcal/mol)
**Organism**	**Protein ID**	**Concentration (μM)**	**Name**	**Concentration (mM)**				
Enterobacter hormaechei	AA4 (MGYG000063141 -00874 – LBD)	65	L-Val	0.2	1.39	0.181	-7.71	0.439
			L-Orn	0.2	1.73	0.323	-3.52	0.307
			L-Met	0.2	2.21	0.414	-8.71	1.12
			L-Lys	0.2	2.26	0.443	-6.5	0.515
			L-Phe	0.2	2.27	0.346	-8.79	0.641
			L-Ile	0.2	2.47	0.331	-9.81	0.768
			L-Ala	0.2	3.5	0.343	-12.9	0.955
			L-Trp	0.35	5.6	1.31	-8.84	1.84
			L-Thr	0.3	15	3.29	-47.1	36.7
			L-Ser	0.4	19.2	10	-71.3	276
			L-Cys	0.3	30	3.16	-68	41.1
			L-Glu	0.3	N/A	N/A	N/A	N/A
Agathobacter faecis	AA5 (MGYG000151212 -00948 – LBD)	65	L-Leu	0.1	0.207	0.034	-12.3	0.411
		62	L-Val	0.15	0.308	0.0642	-9.78	0.494
		65	L-Ile	0.1	0.409	0.109	-18.9	1.58
Ligilactobacillus ruminis ATCC 25644	AA6 (MGYG000210351 -01325 –LBD)	50	L-Arg	2.5	193	17.3	-5.71	0.951
